# Supplementary material for: A novel use of HIV surveillance and court data to understand and improve care among a population of people with HIV experiencing criminal charges in North Carolina 2017–2020
Source: PLoS One. 2025 Mar 27;20(3):e0302767. doi: 10.1371/journal.pone.0302767 (PMC11949325; doi:10.1371/journal.pone.0302767)
Supplement: S1 Table — (PDF) [file pone.0302767.s001.pdf]

S1 Table. Department of public health versus Court record coding of race/ethnicity									
		**Department of Public Health Race/ethnicity coding**							
**Court Race/ethnicity coding**		Black, non-Hispanic	Black, Hispanic	White, Hispanic	White, non-Hispanic	Asia n	American Indian/Alaska native	Other or unknown	**Row totals**
	Black	7226	53	10	47	6	11	0	7353
	White	41	2	58	1742	3	14	0	1860
	Hispanic	7	2	109	11	3	0	1	133
	Other or Unknown	70	6	15	52	21	0	4	168
	**Column totals**	7344	63	192	1852	33	25	5	
