# Supplementary material for: A novel use of HIV surveillance and court data to understand and improve care among a population of people with HIV experiencing criminal charges in North Carolina 2017–2020
Source: PLoS One. 2025 Mar 27;20(3):e0302767. doi: 10.1371/journal.pone.0302767 (PMC11949325; doi:10.1371/journal.pone.0302767)
Supplement: S2 Table — (PDF) [file pone.0302767.s002.pdf]

S2 Table. Baseline characteristics of people with HIV without full follow-up periods (n=2,847)

	n (col%) or median (1st-3rd quartiles)
**Sex**	
Male	2,228 (78.3)
Female	619 (21.7)
**Race**	
White	568 (20.0)
Black	2,204 (77.4)
Hispanic	26 (0.9)
Other or Unknown	49 (1.7)
**Age (years)**	38 (29-50)
**Charge days**	78 (1-257)
**HIV transmission group**	
MSM	1,305 (45.8)
Unknown	771 (27.1)
Heterosexual contact	410 (14.4)
IDU	203 (7.1)
MSM & IDU	138 (4.8)
Other	20 (0.7)

Abbreviations: MSM, men who have sex with men; IDU, injection drug use; IQR interquartile range
