# Supplementary material for: A novel use of HIV surveillance and court data to understand and improve care among a population of people with HIV experiencing criminal charges in North Carolina 2017–2020
Source: PLoS One. 2025 Mar 27;20(3):e0302767. doi: 10.1371/journal.pone.0302767 (PMC11949325; doi:10.1371/journal.pone.0302767)
Supplement: S3 Table — (PDF) [file pone.0302767.s003.pdf]

S3 Table. Association[1] between criminal charge period and duration of unresolved charges NC 2017-2020: Results of multivariable log-binomial model (n=6,427)[2] Analysis including data from all NC counties.

Duration of unresolved charges in days	n (%)	% with viral suppression pre/post charge period	RR of viral suppression in post-charge period compared to pre-charge period
0-99	4,237 (66)	69.8/71.8	1.03 (1.01-1.04)
100-199	984 (15)	72.4/74.3	1.03 (0.90-1.06)
200-299	592 (9)	68.6/72.6	1.06 (1.01-1.10)
300+	604 (10)	64.4/68.1	1.06 (1.01-1.12)

[1] Multivariable log-binomial estimates

[2] Only individuals who contributed person-time to both pre- and post-charge periods and who had a single period of criminal charges were included in model
