# Supplementary material for: A novel use of HIV surveillance and court data to understand and improve care among a population of people with HIV experiencing criminal charges in North Carolina 2017–2020
Source: PLoS One. 2025 Mar 27;20(3):e0302767. doi: 10.1371/journal.pone.0302767 (PMC11949325; doi:10.1371/journal.pone.0302767)
Supplement: S4 Table — (PDF) [file pone.0302767.s004.pdf]

S4 Table. Baseline characteristics of people with and without HIV who charged in criminal court in NC between 2017-2020. Data limited to 26 counties with jail incarceration data available

	Jail Incarcerated and Unincarcerated		Jail Unincarcerated Only		Jail Incarcerated Only	
**Group**	Non-HIV (n=155,745)	HIV (n=1,304)	Non-HIV (n=48,846)	HIV (n=494)	Non-HIV (n=105,597)	HIV (n=989)
	n (col%) or median (1st-3rd quartiles)					
**Sex**						
Male	118,267 (75.9)	1,087 (83.4)	37,781 (77.3)	404 (81.8)	79,399 (75.2)	832 (84.1)
Female	37,478 (24.1)	217 (16.6)	11,065 (22.7)	90 (18.2)	26,198 (24.8)	157 (15.9)
**Race**						
White	72,127 (46.7)	267 (20.5)	20,787 (42.6)	92 (18.6)	51340 (48.4)	214 (21.6)
Black	72,805 (47.1)	1,005 (77.1)	25,556 (52.3)	391 (79.1)	47,997 (45.0)	748 (75.6)
Hispanic	5,582 (3.6)	12 (0.9)	1,415 (2.9)	3 (0.6)	4,177 (3.9)	10 (1.0)
Other or Unknown	3,034 (2.0)	20 (1.5)	1,088 (2.2)	8 (1.6)	2,158 (2.0)	17 (1.7)
**Age (years)**	31 (25-40)	37 (28-49)	31 (25-40)	38 (28-49)	31 (24-40)	37 (28-48)
**Total number of charge periods**						
1	129,870 (84.1)	1,301 (99.8)	35,352 (72.4)	492 (99.6)	94,518 (89.5)	986 (99.7)
2	21,457 (13.9)	3 (0.2)	11,509 (23.6)	2 (0.4)	9,948 (9.4)	3 (0.3)
3 or greater	3,114 (2.0)	0	1,985 (4.1)	0	1,129 (1.1)	0
**Charge days**	139 (56-270)	121 (37-277)	91 (25-40)	65 (2-182)	165 (73-301)	161 (64-309)
**HIV transmission group**						
MSM		506 (38.8)		183 (37.0)		387 (39.1)
Unknown		414 (31.7)		151 (30.6)		319 (32.3)
Heterosexual contact		175 (13.4)		74 (15.0)		125 (12.6)
IDU		125 (9.6)		60 (12.1)		90 (9.1)
MSM & IDU		78 (6.0)		24 (4.9)		63 (6.4)
Other		6 (0.5)		2 (0.4)		5 (0.5)

Abbreviations: NC, North Carolina; MSM, men who have sex with men; IDU, injection drug use; IQR interquartile range.
