# Supplementary material for: A novel use of HIV surveillance and court data to understand and improve care among a population of people with HIV experiencing criminal charges in North Carolina 2017–2020
Source: PLoS One. 2025 Mar 27;20(3):e0302767. doi: 10.1371/journal.pone.0302767 (PMC11949325; doi:10.1371/journal.pone.0302767)
Supplement: S5 Table — (PDF) [file pone.0302767.s005.pdf]

S5 Table. Viral suppression outcome[1] among people with HIV (PWH) in NC with a single criminal charge between 2017-2020: results of multivariable log-binomial model limited to NC counties with available jail incarceration data[2].

	26 counties where jail incarceration data available (n=857)	Unincarcerated during charge period (n=246)	Incarcerated during charge period (n=611)
	**Adjusted RR (95% CI)[3]**	**Adjusted RR (95% CI)[3]**	**Adjusted RR (95% CI)[3]**
**Criminal Charge Group**			
12 months pre charge	Reference		
12 months post charge	0.97 (0.90-1.04)	0.98 (0.89-1.08)	0.98 (0.89-1.07)
**Age (years)**			
18-29	Reference		
30-39	1.11 (0.96-1.28)	0.96 (0.80-1.14)	1.19 (0.98-1.44)
40-49	1.28 (1.10-1.49)	1.01 (0.81-1.26)	1.41 (1.16-1.71)
50-59	1.49 (1.28-1.74)	1.15 (0.94-1.42)	1.63 (1.33-2.00)
60-69	1.26 (0.93-1.71)	0.91 (0.57-1.44)	1.41 (0.96-2.07)
70 and older	NA	NA	NA
**Race/Ethnicity**			
White	Reference		
Black	0.83 (0.73-0.93)	0.85 (0.70-1.03)	0.82 (0.71-0.96)
Hispanic	1.59 (1.35-1.89)	1.59 (1.23-2.05)	1.52 (1.15-2.01)
Other	0.93 (0.60-1.44)	0.81 (0.39-1.69)	1.60 (0.84-1.59)
**Sex**			
Male	Reference		
Female	0.85 (0.72-1.01)	0.92 (0.72-1.18)	0.79 (0.63-0.99)
**HIV Transmission Category**			
MSM	Reference		
Heterosexual	1.00 (0.85-1.88)	1.66 (0.89-1.49)	0.96 (0.77-1.17)
IDU	0.81 (0.67-0.98)	0.93 (0.72-2.00)	0.78 (0.60-1.00)
MSM and IDU	1.02 (0.82-1.26)	1.12 (0.84-1.50)	0.98 (0.77-1.25)
Other	0.99 (0.77-1.00)	0.93 (0.80-1.09)	1.24 (1.06-1.45)

[1] Multivariable log-binomial model estimates, adjusting for all listed variables.[2] Only individuals who contributed person-time to both pre- and post-charge periods and who had a single period of criminal charges were included in model. [3]Adjusted RRs are adjusted for all the variables in the table. Abbreviations: RR, risk ratio; CI, confidence interval; HIV, human immunodeficiency virus; MSM, men who have sex with men; IDU, injection drug use. Definitions: Viral Suppression: <200 copies of HIV RNA per milliliter of blood
