# Supplementary material for: A novel use of HIV surveillance and court data to understand and improve care among a population of people with HIV experiencing criminal charges in North Carolina 2017–2020
Source: PLoS One. 2025 Mar 27;20(3):e0302767. doi: 10.1371/journal.pone.0302767 (PMC11949325; doi:10.1371/journal.pone.0302767)
Supplement: Attachment — Submitted filename: 8_30_24 Response to Reviewers.docx [file pone.0302767.s006.docx]

Dear Reviewers/Editors,

Thank you for your helpful comments and feedback. I have responded to each suggestion below.

Sincerely,

Elizabeth Arant

1. Please ensure that your manuscript meets PLOS ONE's style requirements, including those for file naming.
   a. **Response: I have made the appropriate edits to the headings, figure naming, and citations**
2. Thank you for stating the following financial disclosure:
   a. **Response: I have added the comment about the funding role to the cover letter and have moved the funding statement to the cover letter**

“This work was funded by the National Institute of Allergy and Infectious Diseases at the National Institutes of Health ([NIH] Grant Number T32AI007001 to E.C.A and R01A129731 to D.L.R).  This research was supported by the University of North Carolina at Chapel Hill Center For AIDS Research (CFAR), an NIH funded program P30AI050410.”

Please state what role the funders took in the study.  If the funders had no role, please state: "The funders had no role in study design, data collection and analysis, decision to publish, or preparation of the manuscript."

If this statement is not correct you must amend it as needed.

Please include this amended Role of Funder statement in your cover letter; we will change the online submission form on your behalf.

3. Please provide a complete Data Availability Statement in the submission form, ensuring you include all necessary access information or a reason for why you are unable to make your data freely accessible. If your research concerns only data provided within your submission, please write "All data are in the manuscript and/or supporting information files" as your Data Availability Statement.
   a. **Response: I have altered the data availability statement in the online submission form**
4. We notice that your supplementary figures are uploaded with the file type 'Figure'. Please amend the file type to 'Supporting Information'. Please ensure that each Supporting Information file has a legend listed in the manuscript after the references list.
   a. **Response: I have included a section in the paper for supplementary information with appropriate file titles. I have also uploaded each supplementary table separately**
5. Please include captions for your Supporting Information files at the end of your manuscript, and update any in-text citations to match accordingly. Please see our Supporting Information guidelines for more information: <http://journals.plos.org/plosone/s/supporting-information>.
   a. **Response: supporting tables have captions at the end of the manuscript and I have updated in-text citations with the new table names**
6. Please review your reference list to ensure that it is complete and correct. If you have cited papers that have been retracted, please include the rationale for doing so in the manuscript text, or remove these references and replace them with relevant current references. Any changes to the reference list should be mentioned in the rebuttal letter that accompanies your revised manuscript. If you need to cite a retracted article, indicate the article’s retracted status in the References list and also include a citation and full reference for the retraction notice.
   a. **I have reviewed my list of citations. While no citations were retracted, I did make some errors in my in-text citations and have made those corrections.**


      [Note: HTML markup is below. Please do not edit.]

      Reviewers' comments:

      Reviewer's Responses to Questions

**Comments to the Author**

1. Is the manuscript technically sound, and do the data support the conclusions?

The manuscript must describe a technically sound piece of scientific research with data that supports the conclusions. Experiments must have been conducted rigorously, with appropriate controls, replication, and sample sizes. The conclusions must be drawn appropriately based on the data presented.

Reviewer #1: Yes

Reviewer #2: Yes

2. Has the statistical analysis been performed appropriately and rigorously?

Reviewer #1: Yes

Reviewer #2: Yes

3. Have the authors made all data underlying the findings in their manuscript fully available?

The [PLOS Data policy](http://www.plosone.org/static/policies.action#sharing) requires authors to make all data underlying the findings described in their manuscript fully available without restriction, with rare exception (please refer to the Data Availability Statement in the manuscript PDF file). The data should be provided as part of the manuscript or its supporting information, or deposited to a public repository. For example, in addition to summary statistics, the data points behind means, medians and variance measures should be available. If there are restrictions on publicly sharing data—e.g. participant privacy or use of data from a third party—those must be specified.

Reviewer #1: Yes

Reviewer #2: Yes

4. Is the manuscript presented in an intelligible fashion and written in standard English?

PLOS ONE does not copyedit accepted manuscripts, so the language in submitted articles must be clear, correct, and unambiguous. Any typographical or grammatical errors should be corrected at revision, so please note any specific errors here.

Reviewer #1: Yes

Reviewer #2: Yes

5. Review Comments to the Author

Please use the space provided to explain your answers to the questions above. You may also include additional comments for the author, including concerns about dual publication, research ethics, or publication ethics. (Please upload your review as an attachment if it exceeds 20,000 characters)

Reviewer #1: This is a very interesting paper that uses linked criminal records and HIV surveillance data to examine viral suppression outcomes before and after criminal charges in NC. The linked dataset is very unique, and the analysis addressed an important public health topic. Overall, the paper is clearly written and I did not identify any major flaws. Minor comments for authors to consider:
A result contradicting the original hypothesis has been found: PWH had slightly better viral suppression outcomes after criminal charges than before. This is a very interesting result. The authors included some nice discussion about this. I am wondering is it possible that this association was observed due to any uncontrolled confounder? If so, what confounder do the authors think it could be?

**Response: Thank you for bringing this up. I think the most likely uncontrolled confounder would be the severity of criminal charges, which we did not capture in this analysis. It is likely that those with relatively minor offenses made up the majority of the population of PWH with criminal charges. My thought is that a minor offense had little, if any, effect on viral suppression. Perhaps brief or limited contact with the criminal justice system would serve as prompt for the individual to re-engage in social services that may also improve engagement in HIV care.**

Have the authors considered using the first viral load test after the criminal charges to see if a lower proportion of viral suppression can be found compared to before? A shorter time window may better capture the immediate impact of criminal charges on HIV viral suppression outcomes. People could have recovered from the mental stress related to criminal charges in the next 12 months, especially if most of the charges were for minor offenses.

**Response: I agree with this, and this point was also brought up when I presented this data at our ID grand rounds. We did use the viral load closest to the time period of the end of the charge, so I do think we captured the first data point post-charge period. One challenging aspect is that charge periods can sometime last for several months (charges take a while to resolve), so even the first VL after the charge period may not represent the impact of that first contact with the criminal justice system.**

Reviewer #2: Title: A novel use of HIV surveillance and court data to understand and improve care among a population of people with HIV experiencing criminal charges in North Carolina 2017- 2020

Study summary
The study by Arant et al. investigates the impact of criminal legal system involvement on HIV outcomes among people with HIV (PWH) in North Carolina (NC). Using a linked database of NC court records, jail incarceration records, and state HIV records, the study analyzed data from PWH charged with offenses between January 2017 and February 2020. The study found that while PWH had longer unresolved charge durations, viral suppression rates modestly increased post-charge resolution. Arant et al.’s findings suggest potential benefits of integrating medical-legal partnerships into healthcare delivery for PWH facing detention.

Major strengths of the study
The study was well-designed and conducted, and the report is also well written.

Minor comments
1. In lines 197-199 the authors mention that “When adjusting for demographic and HIV-specific factors, the period following resolution of charges was similarly associated with an increased risk of viral suppression.” Since viral load suppression is a positive outcome, I suggest the authors use "likelihood" or "odds" instead of "risk" which is associated with a negative outcome.

**Response: Thank you for pointing this out. I have adjusted the wording in the text**

6. PLOS authors have the option to publish the peer review history of their article ([what does this mean?](https://journals.plos.org/plosone/s/editorial-and-peer-review-process#loc-peer-review-history)). If published, this will include your full peer review and any attached files.


If you choose “no”, your identity will remain anonymous but your review may still be made public.


**Do you want your identity to be public for this peer review?** For information about this choice, including consent withdrawal, please see our [Privacy Policy](https://www.plos.org/privacy-policy).

Reviewer #1: No

Reviewer #2: **Yes:** Lweendo Muchaili
